# Combination of Clptm1L and TMEM207 Expression as a Robust Prognostic Marker in Oral Squamous Cell Carcinoma

**DOI:** 10.3389/froh.2021.638213

**Published:** 2021-03-29

**Authors:** Kimika Hano, Kiichi Hatano, Chiemi Saigo, Yusuke Kito, Toshiyuki Shibata, Tamotsu Takeuchi

**Affiliations:** ^1^Department of Oral and Maxillofacial Surgery, Gifu University Graduate School of Medicine, Gifu, Japan; ^2^Department of Pathology and Translational Research, Gifu University Graduate School of Medicine, Gifu, Japan

**Keywords:** OSCC, prognosis, TMEM207, CLPTM1L, ER-stress, WWOX

## Abstract

Overexpression of Cleft Lip and Palate Transmembrane 1-Like (Clptm1L) confers cancer cell survival through the endoplasmic reticulum (ER) stress survival signaling pathway, while TMEM207 impairs the tumor suppressor function of WW domain containing oxidoreductase (WWOX), which sensitizes cancer cells to ER stress-induced apoptosis. In the present study, we examined whether these two ER stress-related proteins, Clptm1L and TMEM207, could be prognostic markers in oral squamous cell carcinoma (OSCC). Immunohistochemical staining using specific antibodies to Clptm1L or TMEM207 revealed that 31 of 89 tissue specimens exhibited concomitant expression of Clptm1L and TMEM207 at the cancer invasion front. A Kaplan–Meier plot of the patient survival curve followed by a log-rank test revealed that the coexpression of Clptm1L and TMEM207 was significantly associated with poor outcome in patients with OSCC (*P* = 0.00252). Coexpression of Clptm1L and TMEM207 was closely related to lymph node metastasis (*P*=0.000574). Both univariate and multivariate analyses demonstrated that coexpression of Clptm1L and TMEM207 predicted the poor prognosis of the patients with OSCC. The present study indicated that the double positive Clptm1L and TMEM207 immunoreactivity was closely related to lymph node metastasis with prognostic value in patients with OSCC.

## Introduction

The incidence of oral squamous cell carcinoma (OSCC) has increased in the recent years. Despite progress in chemotherapy and radiotherapy, the prognosis of advanced OSCC remains unfavorable [1]. This is contrary to the improved prognosis of patients with other cancers, such as breast cancer, lymphoma, lung cancer, and gastrointestinal cancers, which has been greatly improved by recent molecular targeted therapies. To develop new molecular targeting therapies with regard to OSCC, there is a need to clarify the pathobiological mechanism, especially the crucial point of signaling cascade in oral squamous carcinogenesis.

The endoplasmic reticulum (ER) is a critical compartment for folding of the secretory, plasma membrane, and various organelle proteins [2]. Under various conditions, because of ER stress, protein folding is impaired, leading to the accumulation of misfolded proteins in the ER [3]. The accumulation of these misfolded proteins is harmful to the cells, and prolonged ER stress typically leads to apoptosis [4]. Therefore, cancer cells are more dependent on stress support survival pathways [5], which could be a good target in molecular therapy.

Cleft Lip and Palate Transmembrane 1-Like (Clptm1L) was first isolated by screening cisplatin resistance-related genes [6]. Subsequently, several studies have demonstrated that Clptm1L is involved in lung carcinogenesis [7–10]. Notably, Clptm1L interacts with GRP78, which mediates ER stress-induced Akt signaling for ER stress survival in pancreatic adenocarcinoma cells [11].

TMEM207 is localized to the ER and binds to the tumor suppressor molecule, WW domain containing oxidoreductase (WWOX), through its PPxY domain [12]. As a result, TMEM207 inhibits the various tumor suppressor function of WWOX, leading to the induction of ER stress-related apoptosis in cancer cells [13, 14]. Notably, the tumor suppressor function of WWOX is dependent on the expression level of GRP78 in ovarian cancer cells [15].

In this study, we aimed to determine the prognostic value of concomitant expression of Clptm1L and TMEM207 in OSCC. Here, we show the robust correlation of Clptm1L and TMEM207 expression with lymph node metastasis and poor prognosis in patients with OSCC.

## Materials and Methods

### Patient Tissue Specimens

For this retrospective study, we collected data of all surgically treated patients primarily diagnosed with OSCC. Archived pathological tissue specimens from 89 OSCC cases were used in this study. The present study was conducted in accordance with the ethical standards of the Helsinki Declaration in 1975. The use of tissue samples and review of the clinical records were performed according to protocols approved by the Institutional Review Board of the Gifu University Graduate School of Medicine (specific approval number: 28-524).

### Antibodies and Immunohistochemical Staining

All tissue specimens were obtained surgically, fixed in 10% buffered formalin, and embedded in paraffin. Tissue sections representing the deepest invasion site of each case were immunohistochemically stained. The rabbit conventional antibody to Clptm1L (dilution 1:100, cat no. NBP1-84378) was obtained from Novus Biologicals (Littleton, CO, USA). Generation and characterization of a murine monoclonal antibody recognizing the synthetic peptide VNYNDQHPNGW (a.a. 40–50 of TMEM207) described previously [12], was employed in this study. Anti-TMEM207 antibody was used at 5 μg/ml for immunohistochemical staining. Control antibodies were used as previously described [12, 15]. The tissue sections deparaffinized, incubated with normal goat serum, and incubated with antibodies for overnight at 4°C. After washing, the tissues were immunostained with antibodies using the ImmPRESS™ polymerized reporter enzyme staining system (Vector Laboratories, Inc., Burlingame, CA, USA) as previously reported [15].

We also performed double immunohistochemical staining of twenty cases using a biotin-free polymer detection kit (MACH 2, Biocare Medical, Walnut Creek, CA, USA) according to the manufacturer's protocol.

### Evaluation of Immunohistochemical Staining and Statistical Analysis

We evaluated the immunohistochemical staining results as a percentage of immunoreactivity in OSCC cells. The fraction of positive cells stained at the cancer invasion front was scored after examining three high-power fields (400×) per tissue section for each case. The staining was considered negative if <10% of invasive cancer cells exhibited immunoreactivity, and positive if over 10% did. One experienced pathologist read the sections without knowing the pathological grade and clinical data.

Curves for overall survival (OS) were drawn using the Kaplan–Meier method, and differences in survival rates were compared using the log-rank test. The relationship between clinicopathological parameters was examined using the chi-square test. Univariate and multivariate analyses of the Cox proportional hazard model were also used to determine the significant prognostic factors of OS. A *P* < 0.05 was considered statistically significant.

## Results

### Clptm1L and TMEM207 Expressions in OSCC Tissue Specimens in Relation to Prognosis

**Figure 1:**
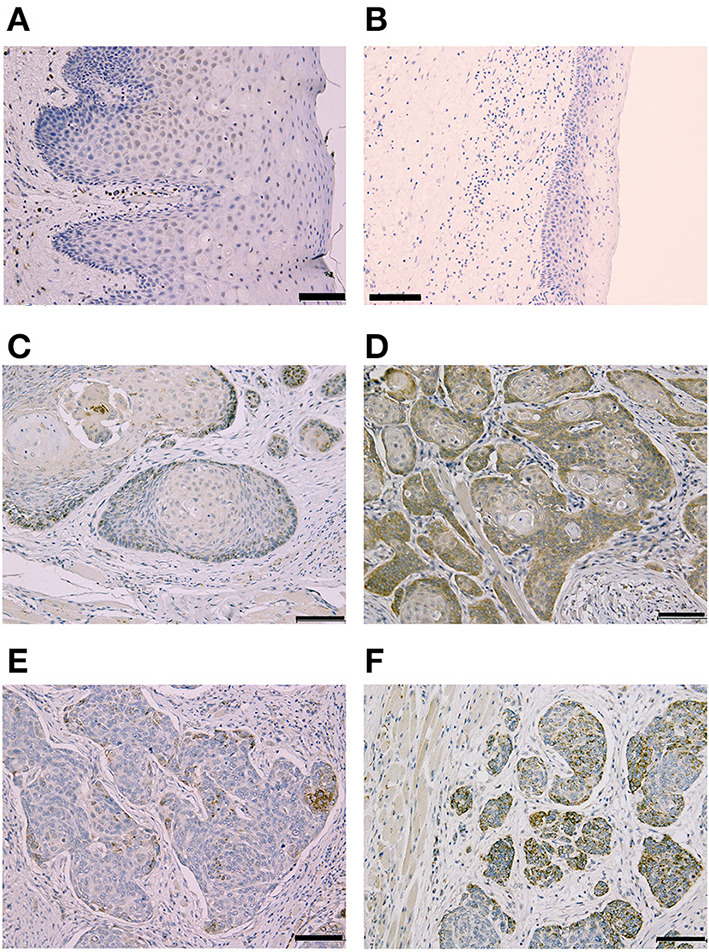
Representative Immunohistochemical staining according to Cleft Lip and Palate Transmembrane 1-Like (Clptm1L) and TMEM207 immunoreactivity at the invasion front of oral squamous cell carcinoma (OSCC). Weak Clptm1L or TMEM207 immunoreactivity was found in non-tumorous oral epithelial cells **(A,B)**, respectively. In contrast, Clptm1L or TMEM207 immunoreactivity was found in many invasive OSCC cells. Representative positive Clptm1L immunoreactivity in tissue specimens from patients with favorable and poor prognosis is shown in **(C,D)**, respectively. TMEM207 immunoreactivity in tissue specimens from patients with favorable and poor prognosis is shown in **(E,F)**, respectively. Note the robust Clptm1L and TMEM207 immunoreactivity in **(D,F)**.

**Figure 2:**
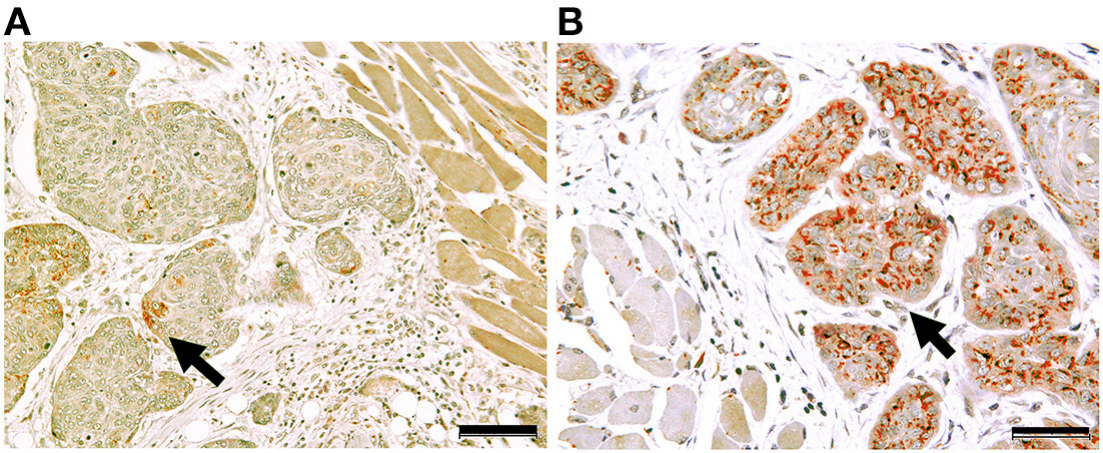
Concomitant expression of Cleft Lip and Palate Transmembrane 1-Like 1 (Clptm1L) and TMEM207 in OSCC cells. Double immunohistochemical staining showed that the Clptm1L, Cleft Lip, and Palate Transmembrane 1-Like (Clptm1L) immunoreactivity (brown color) and TMEM207 immunoreactivity (red color) were co-localized in OSCC cells during cancer invasion in a case of poor outcome **(B)**. In contrast, little Clptm1L or TMEM207 immunoreactivity was found in a case with a favorable outcome **(A)**. Arrows indicate positive staining. Bar indicates 50 μm.

Clinicopathological features of OSCC cases are shown in Table 1. Representative immunohistochemical staining is shown in Figure 1. Non-cancerous oral stratified squamous epithelium exhibited little Clptm1L or TMEM207 immunoreactivity. In contrast, 50 and 40 of 89 OSCC tissue specimens exhibited Clptm1L or TMEM207 immunoreactivity, respectively. Notably, Clptm1L and TMEM207 expressions were significantly related to each other (Table 2, *P* = 0.0002). We considered 31 tissue specimens as double positive for Clptm1L and TMEM207. Concomitant expression of Clptm1L and TMEM207 expression was confirmed by double immunohistochemical staining (Figure 2).

**Table 1:** Summary of the demographic and clinicopathological characteristics of patients with OSCC.

**Characteristic**	**No. of patients (%)**
Gender	
Male	43 (48)
Female	46 (52)
Age (years)	
50<	15 (17)
50≧	74 (83)
Tumor location	
TON	37 (42)
LG	24 (27)
BM	13 (15)
UG	9 (10)
FOM	4 (4)
HP	2 (2)
Histology of SCC	
Well-differentiated	60 (67)
Moderately-differentiated	23 (26)
Poorly-differentiated	6 (7)
Tumor T status	
T1	25 (28)
T2	43 (48)
T3	11 (13)
T4	10 (11)
Tumor N status	
N0	53 (60)
N1	17 (19)
N2	11 (12)
N3	8 (9)
Tumor M status	
M = 0	89 (100)
M = 1	0 (0)
Stage (UICC 8th ED)	
I	19 (21)
II	26 (29)
III	15 (17)
IV	29 (33)
Radiotherapy/Chemotherapy	
None	59 (66)
Radiotherapy only	1 (1)
Chemotherapy only	11 (13)
Radiotherapy+Chemotherapy	18 (20)
Survival status	
Alive	59 (66)
Deceased	30 (34)
Disease-associated death	22 (25)

*T and N data based on pathology examination. The time for survival is 5-year*.

*TON, tongue; LG, lower gum; BM, buccal mucosa; UG, upper gum; FOM, floor of mouth; HP, hard palate*.

**Table 2:** Relation of Clptm1L and TMEM207 expression in OSCC.

**TMEM207 positive**	**TMEM207 negative**
Clptm1L positive	30
Clptm1L negative	19

*Clptm1L and TMEM207 expression is significantly related to each other*.

*(P = 0.0002)*.

**Figure 3:**
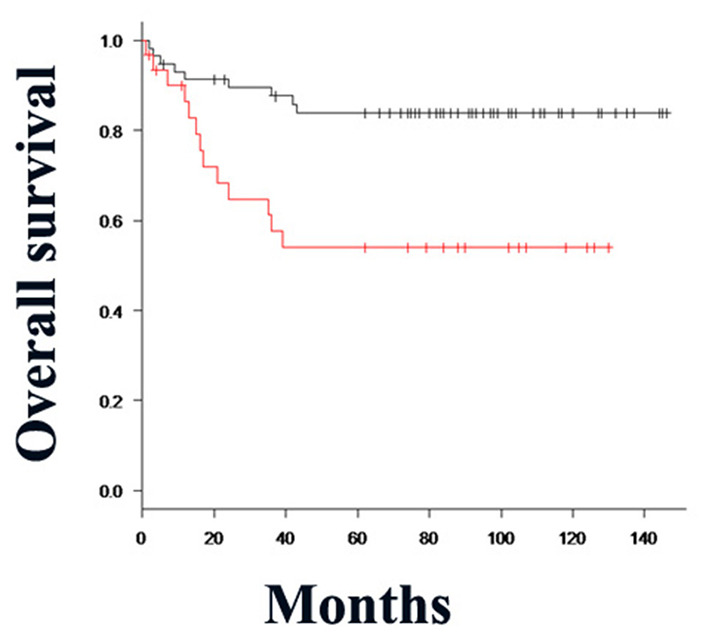
Kaplan–Meier method and differences in the survival rates were compared using the log-rank test for univariate survival analysis. The overall survival rate of patients in the Clptm1L and TMEM207 co-expressed group (red line) was significantly lower than that of patients in the Clptm1L or TMEM20 negative group (black line) (*P* = 0.00252).

Double Clptm1L and TMEM207 immunoreactivity was significantly related to pT (T1/2 vs. T3/T4) (*P* = 0.0148), pN stage (*P* = 0.000574), and smoking habits (*P* = 0.0032) of patients with OSCC (Table 3). A Kaplan–Meier plot of the patient survival curve followed by log-rank test revealed that the concomitant expression of Clptm1L and TMEM207 was significantly related to poor outcome in patients with OSCC (*P* = 0.00252) (Figure 3). In the univariate analysis, nodal metastasis (*P* = 0.0005), UICC stage (I + II vs. III + IV) (*P* = 0.0089), and double positive for Clptm1L and TMEM207 (*P* = 0.0046) were significantly related to poor outcome of the patients (Table 4). Multivariate Cox proportional hazards regression analysis indicated that double positive for Clptm1L and TMEM207 expression yielded a hazard ratio of death of 2.466 (95% confidence limit, 1.085–6.386, *P* = 0.032) in the OSCC series.

**Table 3:** Correction between Clptm1L and TMEM207 immunoreactivity and clinicopathological factors.

**Clpmt1L2.TMEM207**			
**Immunoreactivity**	**Double positive**	**Others**	***P-*** **value**
Gender			
Male	17	26	
Female	14	32	0.384
Age			
<50	6	9	
≧50 years	25	49	0.768
Histology of SCC			
Well-differentiated	22	38	0.643
Moderately-poorly differentiated	9	20	
Stage (UICC 8th Ed)			
I+II	10	35	
III+IV	21	23	0.0148
Lymph node metastasis			
No	11	43	
Yes	20	15	0.000574
Smoking			
No	16	43	0.0032
Yes	15	15	

**Table 4:** Cox multivariate analysis in OSCC.

	**Hazards ratio (95% CI)**	***P-*value**
**UNIVARIATE**		
Gender	1.799 (0.7681, 4.214)	0.1762
Age (<50 vs. ≧50 years)	1.028 (0.9954, 1.061)	0.09317
Histological differentiation	1.065 (0.4339, 2.612)	0.8913
(Well-differentiated vs. moderately and poorly differentiated)		
UICC stage (I + II vs. III + IV)	3.508 (1.37, 8.983)	0.00891
Lymph node metastasis (no vs. yes)	3.472 (1.455, 8.288)	0.005042
Double positive of Clpmt1L and TMEM207		
Vs. Others	3.431 (1.462, 8.048)	0.0046
**MULTIVARIATE**		
UICC stage (I + II vs. III + IV)	2.721 (1.023, 7,234)	0.045
Clpmt1L2.TMEM207	2.466 (1.085, 6.386)	0.032

## Discussion

In the present study, we found that Clptm1L and TMEM207 were significantly related to smoking habits in patients with OSCC. Several studies have reported that cigarette smoke induces ER stress in normal and various malignant tumor cells [16]. It is likely that overexpression of Clptm1L and TMEM207 may promote oral squamous cell carcinogenesis by conferring resistance to cigarette smoke-associated ER stress. Alternatively, cigarette smoking-associated ER stress might induce Clptm1L and TMEM207 expression in OSCC cells. Further studies are needed to unravel the relationship between cigarette smoke and Clptm1L and/or TMEM207 expression in OSCC cells.

Using the Kaplan–Meier (log rank) test, the *P-*value for the difference between concomitant expression of Clptm1L-TMEM207 and others = 0.00252 for OS time Notably, Multivariate Cox's regression analysis unraveled that concomitant expression of Clptm1L-TMEM207 was an independent worse prognosis value (*P* = 0.032) (Table 4).

The exact molecular mechanism, which results in close relation of concomitant Clptm1L-TMEM207 expression and nodal metastasis remains unclear. Interestingly, overexpression of GRP78 increases lymph node metastasis in various cancers [17–19]. GRP78 might be a key molecule associated with high lymph node metastasis rate in Clptm1L and TMEM207 co-expressed OSCC.

Oliveira and Ribeiro-Silva classified immunohistochemical biomarkers with prognostic significance as cell cycle, apoptosis, angiogenesis, cell adhesion, and matrix degradation-related molecules in OSCC [20]. In contrast, the present study revealed that two ER stress-related proteins, Clptm1L and TMEM207, could also be a good biomarker to predict poor outcomes in patients with OSCC. We believe that molecules which help OSCC cells evade ER stress could also be a target of molecular therapy in patients with OSCC.

In conclusion, the present study demonstrated that the expression of two ER stress-related proteins, Clptm1L and TMEM207 immunoreactivity was closely related to lymph node metastasis with prognostic value in patients with OSCC.

## Data Availability Statement

The raw data supporting the conclusions of this article will be made available by the authors, without undue reservation.

## Ethics Statement

The studies involving human participants were reviewed and approved by Institutional Review Board of the Gifu University Graduate School of Medicine (specific approval number: 28-524). Written informed consent for participation was not required for this study in accordance with the national legislation and the institutional requirements.

## Author Contributions

KHan and KHat carried out the scoring, performed the statistical analysis, and interpreted the results. CS and YK designed the project and wrote the manuscript. TS contributed to the manuscript text. TT designed and supervised the project. All authors critically revised the manuscript.

## Conflict of Interest

The authors declare that the research was conducted in the absence of any commercial or financial relationships that could be construed as a potential conflict of interest.

## Abbreviations

Clptm1L: Cleft Lip and Palate Transmembrane 1-Like
ER: endoplasmic reticulum
WWWOX: WW domain containing oxidoreductase
OSCC: oral squamous cell carcinoma
OS: overall survival
TON: tongue
LG: lower gum
BM: buccal mucosa
UG: upper gum
FOM: floor of mouth
HP: hard palate.
